# Traumatic Arteriovenous Fistula of the Scalp in the Left Temporoparietal Region with Intra- and Extracranial Blood Supply

**DOI:** 10.1155/2016/8671472

**Published:** 2016-01-18

**Authors:** Feng Zheng, Herbert Augustus Pitts, Roland Goldbrunner, Boris Krischek

**Affiliations:** ^1^Department of Neurosurgery, University Hospital of Cologne, 50937 Cologne, Germany; ^2^Department of Anatomical and Cellular Pathology, Prince of Wales Hospital, Chinese University of Hong Kong, Hong Kong

## Abstract

Traumatic AVF of the scalp is a rare abnormal vascular disease. It is defined as a communication between the high flow arterial system and the low flow venous network, which directly connects the arterial feeding vessels of the scalp and the draining veins without an intervening capillary bed. The superficial temporal artery (STA) was involved in 90% of the cases, and 71% of the patients only had one dominant feeding STA. Here, we report the case of a rare large traumatic arteriovenous fistula (AVF) of the scalp that is fed by intra- and extracranial blood supply. The clinical and radiological features are presented, and the possible pathogenesis and surgical technique are discussed.

## 1. Introduction

Traumatic AVF of the scalp is rare vascular disease [1, 2] and is defined as a communication between the high flow arterial system and the low flow venous system. It is a direct connection between the arterial feeding vessels of the scalp and the draining veins without an intervening capillary bed [2]. It presents as a disfiguring pulsatile lesion associated with various clinical manifestations, such as bruits, tinnitus, local pain, headaches, epilepsy, hemorrhage, and scalp necrosis [3, 4].

In 90% of the patients, superficial temporal artery (STA) was involved, and in 71% of the cases there was one dominant feeding STA [5]. Several methods, such as open surgical removal, ligation of the feeding arteries, trans-arterial and trans-venous embolization, and intralesional injection of sclerosant, have been used to treat these cases [1, 6].

Here, we present a case of a large sized fistula fed by a complex network of feeding vessels that involved both intra- and extracranial blood supply, which, to the best of our knowledge, has never been reported previously. According to the angioarchitecture and presentation of the case, and for economic reasons, we chose straight-forward surgical treatment of the AVF over a pure endovascular treatment or in combination with surgery.

## 2. Case Report

**Figure 1:**
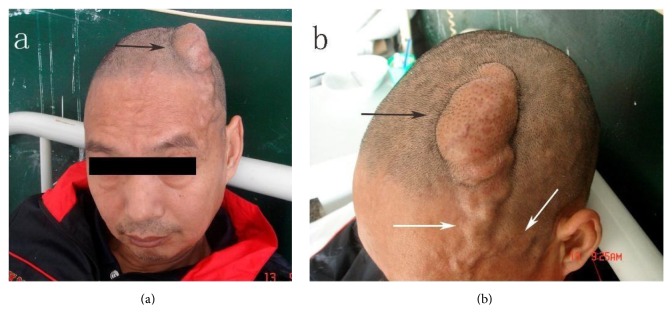
Macroscopic picture of the patient's head. (a) The black arrow points to a large mass in the left temporoparietal area. (b) The white arrow is pointing to the left tortuous STA; the black arrow shows the vascular mass, which was pulsatile, and presented a clear wind-like murmur on auscultation. STA: superficial temporal artery.

**Figure 2:**
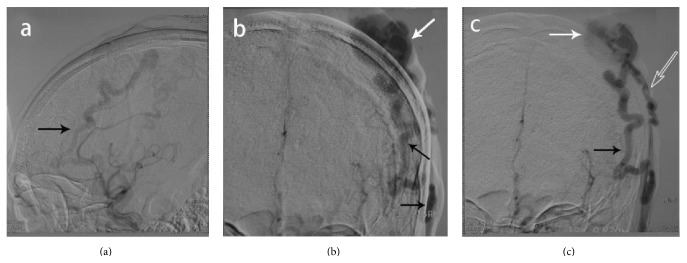
Digital subtraction angiography of the left ECA. (a) Lateral projection, early imaging, black arrow shows the left tortuous STA. (b) Anterior-posterior projection, black arrow shows the left STA which is thickened and tortuous supplies of blood, and the vascular mass is huge; the white arrow shows the point of the fistula. (c) Delayed imaging, black arrow shows the tortuous left STA, white arrow shows the vascular mass, and the hollow arrow shows the left subcutaneous drainage vein. ECA: external carotid artery; STA: superficial temporal artery.

**Figure 3:**
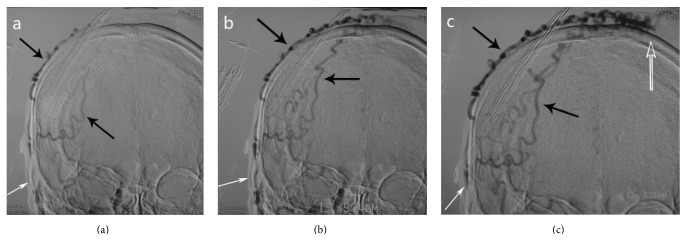
Digital subtraction angiography of the right ECA. (a) Anterior posterior projection, early imaging, white arrow shows the right STA; black arrow shows the tortuous branches of the right STA. ((b) and (c)) Anterior posterior projection, tortuous branches of the right STA supply the fistula; the hollow arrow indicates the point of the fistula. ECA: external carotid artery; STA: superficial temporal artery.

**Figure 4:**
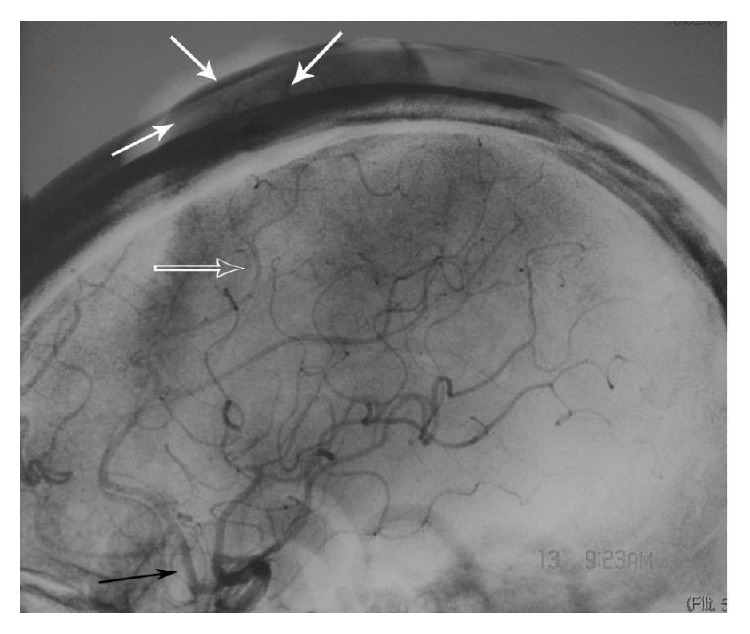
Digital subtraction angiography of left ICA (lateral projection). The black arrow shows the ACA, the hollow arrow shows a branch of the pericallosal artery, and the white arrows show the supply to the traumatic fistula. ICA: internal carotid artery; ACA: anterior cerebral artery.

A 57-year-old man was diagnosed with paranoid schizophrenia at the age of 20. Eight years prior to presentation in the emergency department, the patient sustained a fist blow to the top of the left temporoparietal region, in a conflict with another patient during treatment in a psychiatric institution. Initially, after the injury, a thumbnail-sized mass developed. It increased in size with time. The patient was therefore hospitalized by his guardian. At presentation an irregular hemispherical mass measuring 8 × 5 × 3 cm was noted in the left temporoparietal area (Figure 1); this mass extended to the forehead and the bottom of the left temple in an earthworm-like form and connected with tortuous vessels from both temporal parts of his head, which were pulsatile. The hemispherical mass was soft and mildly tender and had a moderately elevated temperature. A clear wind-like murmur was heard on auscultation of the mass. Examinations of the heart and lungs and other systemic examinations showed no abnormalities, and laboratory test results were also within normal range. The patient underwent a digital subtraction angiography: (1) the left superficial temporal artery (STA) was thickened and tortuous, and there was a large vascular mass at its end (Figure 2). (2) The right STA was also thickened and tortuous and was also supplying blood to the vascular mass (Figure 3). (3) The left anterior cerebral artery (ACA) was also leading blood to the fistulous mass (Figure 4). (4) The vascular mass drained through both the external jugular veins, which also appeared thickened and tortuous (Figure 4). On the basis of these findings, the condition was diagnosed as a traumatic AVF of the scalp in the left temporal parietal area, supplied by both STAs and the left ACA.

After obtaining informed consent from his guardian, the AVF was resected under general anesthesia. In order to decrease the intraoperative bleeding, we first temporarily ligated the proximal branch of the feeding vessels (both STAs) twice, by suturing through the skin with a silk suture; then a 25 cm long horseshoe-shaped scalp flap including the galea was incised around the mass and pedicled towards the temporal region. Subsequently, the feeding arteries and draining veins were isolated and sequentially ligated with the exception of the feeding arteries within the periosteum, which as we suspected arose from intracranially (according to the angiography, they were deriving from the ACA). The periosteal feeding vessels were cut superficially after coagulation. The overlying arteriovenous fistulous mass was then removed. By leaving the periosteum that contained stumps of the feeding vessels which originated from the left ACA, we prevented retraction of the stumps and a possible subsequent intracranial bleeding. After resection of the AVF, we meticulously cauterized minor bleeding sites with the bipolar coagulation forceps, particularly on and around the deliberately left periosteum and then released the previous temporary ligations around the STAs. Lastly, the wound was closed in a multilayered fashion. The surgery was uneventful, with blood loss of less than 50 mL. The pathological report revealed many irregular blood-supplying arteries with large diameters and thickened walls that were interwoven to form a mass and arteriovenous fistula. A postoperative CT scan showed no intracranial hemorrhage. The wounds healed nicely, and the shape of the head appeared normal. Furthermore, there was no recurrence in the 1-year follow-up.

## 3. Discussion

Wardrop first reported this disease in 1827 [7]. Subsequently, traumatic AVF of the scalp was considered to be supplied by either the STA or the STA in combination with the middle meningeal artery, without the involvement of the internal carotid artery (ICA) [1, 2, 8–10]. To our knowledge, this case is the largest reported, being supplied by both STAs and branches of the ICA.

It is generally accepted that scalp arteriovenous fistula may be of either congenital or traumatic lesion. Most congenital lesions become symptomatic in the third decade of life (range: three months to 59 years of age) [11]. Matsushige et al. reported a case of scalp arteriovenous malformation; vascular endothelial growth factor is expressed by these lesions and is responsible for continuous growth [12]. Two mechanisms have been cited in explaining the pathophysiology of traumatic arteriovenous fistulae in the scalp. The first mechanism, a simultaneous laceration of the artery and the adjacent vein, leads to formation of the fistula. The second mechanism begins with the rupture of vasa vasorum in the artery wall. Proliferation of endothelial cells from the damaged vasa vasorum then forms numerous small vessels, leading to vascular communication channels between artery and vein [13, 14]. In the case of the patient described here, we assume there was an initial damage to the intracranial vessels and simultaneous local skull fracture. This led to an extrorse proliferation of the newly formed small vessels, which connected intra- and extracranial vessels and in turn fed the fistula which had previously been formed by both STAs after the trauma.

The disease is characterized by four factors: a pulsating mass on the head, thickening of the vasculature morphologically and on angiography, pulsatile sounds on auscultation that will decrease and eventually disappear when the main feeding arteries are depressed, and draining veins. Angiography is the gold standard diagnostic tool to highlight the angioarchitecture [2].

For economic reasons, we opted for conventional surgical excision, which is cost efficient and does not bear the risk of cosmetic irregularities after interventional injection/placement of embolic material in the subcutaneous tissue [1, 2, 15]. During resection of the AVF, we deliberately left the local periosteum in order to avoid intracranial bleeding which may occur due to the retraction of the stumps of ACA.
